# A Quantitative and Narrative Evaluation of Goodman and Gilman’s Pharmacological Basis of Therapeutics

**DOI:** 10.3390/pharmacy8010001

**Published:** 2019-12-20

**Authors:** Brian J. Piper, Alexandria A. Alinea, John R. Wroblewski, Sara M. Graham, Daniel Y. Chung, Livia R. M. McCutcheon, Melissa A. Birkett, Steven S. Kheloussi, Vicky M. Shah, John L. Szarek, Qais K. Zalim, John A. Arnott, William A. McLaughlin, Pamela A. Lucchesi, Kimberly A. Miller, Gabi N. Waite, Michael Bordonaro

**Affiliations:** 1 Department of Medical Education, Geisinger Commonwealth School of Medicine, Scranton, PA 18510, USA; aalinea@som.geisinger.edu (A.A.A.); JWroblewski@som.geisinger.edu (J.R.W.); SGraham@som.geisinger.edu (S.M.G.); dyc4321@gmail.com (D.Y.C.); JSzarek@som.geisinger.edu (J.L.S.); jarnott@som.geisinger.edu (J.A.A.); wmclaughlin@som.geisinger.edu (W.A.M.); KMiller02@som.geisinger.edu (K.A.M.); GWaite@som.geisinger.edu (G.N.W.); mbordonaro@som.geisinger.edu (M.B.); 2 Center for Pharmacy Innovation and Outcomes, Geisinger Precision Health Center, Forty Fort, PA 18704, USA; 3 Department of Pharmacy Practice, Wilkes University, Wilkes-Barre, PA 18702, USA; livia.mccutcheon@wilkes.edu (L.R.M.M.); steven.kheloussi@wilkes.edu (S.S.K.); vicky.shah@wilkes.edu (V.M.S.); 4 Department of Psychology, Southern Oregon University, Ashland, OR 97520, USA; birkettm@sou.edu; 5 Department of Psychiatry, Wright Center for Graduate Medical Education, Scranton, PA 18503, USA; zalimq@thewrightcenter.org; 6 School of Medicine, New York Medical College, Valhalla, NY 10595, USA; plucches@nymc.edu

**Keywords:** education, ethics, pharmacy, pharmacology, textbook

## Abstract

Goodman and Gilman’s *The Pharmacological Basis of Therapeutics* (_GG_PBT) has been a cornerstone in the education of pharmacists, physicians, and pharmacologists for decades. The objectives of this study were to describe and evaluate the 13^th^ edition of _GG_PBT on bases including: (1) author characteristics; (2) recency of citations; (3) conflict of interest (CoI) disclosure; (4) expert evaluation of chapters. Contributors’ (N = 115) sex, professional degrees, and presence of undisclosed potential CoI—as reported by the Center for Medicare and Medicaid’s Open Payments (2013–2017)—were examined. The year of publication of citations was extracted relative to Katzung’s *Basic and Clinical Pharmacology* (_Kat_BCP), and DiPiro’s *Pharmacotherapy: A Pathophysiologic Approach* (_DiP_PAPA). Content experts provided thorough chapter reviews. The percent of _GG_PBT contributors that were female (20.9%) was equivalent to those in _Kat_BCP (17.0%). Citations in _GG_PBT (11.5 ± 0.2 years) were significantly older than those in _Kat_BCP (10.4 ± 0.2) and _DiP_PAPA (9.1 ± 0.1, *p* < 0.0001). Contributors to _GG_PBT received USD 3 million in undisclosed remuneration (Maximum author = USD 743,718). In contrast, _DiP_PAPA made CoI information available. Reviewers noted several strengths but also some areas for improvement. _GG_PBT will continue to be an important component of the biomedical curriculum. Areas of improvement include a more diverse authorship, improved conflict of interest transparency, and a greater inclusion of more recent citations.

## 1. Introduction

Educator and pharmacologist Louis S. Goodman (1906–2000) completed his undergraduate degree at Reed College and his MD at the University of Oregon Medical School in Portland, Oregon. Goodman and Alfred Gilman, his colleague and collaborator on nitrogen mustard investigations, published the first edition of *Pharmacological Basis of Therapeutics* (_GG_PBT) in 1941. Louis’s son Alfred G (Goodman) Gilman (1941–2015) received a Nobel Prize for his work on signal transduction and served in various editorial capacities for the 5^th^ to 10^th^ editions. The first reviewer was “delirious in his appraisal of the book” and anticipated it would become the standard text in pharmacology [1]. The eighteen-hundred-page 2^nd^ edition, published in 1956, was referred to as encyclopedic and indispensable [2]. A reviewer noted that “all other related books seem to pale by comparison” [3]. An evaluation of the 6^th^ edition published in 1980 commended the extensive bibliography, but was more measured and noted that, although “this book is recommended to all those who prescribe drugs”, _GG_PBT had become “too large to be used by medical students as a routine textbook” [4]. Hastings and Long referred to _GG_PBT as the “blue bible of pharmacology” and the “gold standard” [5]. Rosenberg warmly commended this reference for dentistry and anesthesiology [6]. However, despite the book’s esteemed and authoritative status, a subsequent reviewer alluded to one chapter where the text had “barely kept up” with the rapid pace of new therapeutic developments. Casavant also noted the omission of twenty of twenty-six newly approved medications in the 10^th^ edition [7]. The paucity of female authors and absence of potential conflict of interest disclosure were concerns expressed about the 12^th^ edition published in 2012 [8].

An under-representation of females as authors has been identified in different fields and types of publications. Female first authorship in six, high-impact, general medical journals increased from 27% in 1994 to 37% in 2014 [9]. Senior authorship by women in cardiology journals doubled from 6% in 1996 to 12% in 2016 [10]. Textbook authorship, which often involves large teams, is highly variable and ranged from half (53%) female for an advanced pharmacy textbook—the 2014 9^th^ edition of DiPiro’s *Pharmacotherapy: A Pathophysiological Approach* (_Dip_PAPA) [11]—to one out of seven (14%) in Yagiela’s *Pharmacology and Therapeutics for Dentistry* [8,12].

Conflicts of interest (CoIs) were defined as “a set of circumstances that creates a risk that professional judgment or actions regarding a primary interest will be unduly influenced by a secondary interest” [13]. The transparency of CoI has become increasingly ubiquitous for primary sources [14] including clinical trials [15], undergraduate medical education [16], continuing medical education [17], point of care computerized sources [18], meta-analyses [19], and clinical practice guidelines [20]. The US Physician Payments Sunshine Act of 2010 required that all compensation (≥$10) to doctors of medicine, osteopathy, dentistry, dental surgery, podiatry, optometry, and chiropractic medicine (i.e., PharmDs, PhDs, PAs, and NPs were not covered although this subsequently changed for PAs and NPs) from manufacturers of drugs and medical devices be reported to the Centers for Medicare and Medicaid Services (CMS), and made available on its public website. Disclosure of CoI was provided in the preface to a psychopharmacology textbook [21] and in _Dip_PAPA but this practice is currently uncommon. The database, ProPublica’s Dollars for Docs (PDD), originally covered only fifteen pharmaceutical companies. Using the first generation of this database, one can see that the authors and editors of four biomedical textbooks had received USD 2.4 million, primarily for speaking and consulting, which was undisclosed to readers. One-quarter of the contributors to _GG_PBT, 12^th^ edition, had an undisclosed patent [8].

Here, we extend upon earlier educational research [8,12] by assessing the most recent (2018, 13^th^ edition) of _GG_PBT [22]. This includes quantitative descriptive measures of author characteristics (e.g., education, sex), a citation analysis relative to other pharmacology (_Kat_BCP) and pharmacotherapy (_DiP_PAPA) textbooks, and characterizing the presence of potential CoIs using more comprehensive databases. Content experts also provided detailed assessments in their specialty areas.

## 2. Materials and Methods

### 2.1. Procedures

The contributors page of the 13^th^ edition of Goodman and Gilman’s *The Pharmacological Basis of Therapeutics* (_GG_PBT) [22] was consulted to obtain the professional degrees of authors and editors (N = 115). Sex was determined using a Google search or consulting the National Provider Identifier (https://www.cms.gov/Regulations-and-Guidance/Administrative-Simplification/NationalProvIdentStand/). The presence of CoI was deemed exempt by the Wright Center Institutional Review Board and evaluated with two publicly available databases—the Center for Medicare and Medicaid Service’s Open Payments (_CMS_OP), and ProPublica’s Dollars for Docs (_PP_DD). These databases differ slightly with respect to when potential CoI information is made available and to details (e.g., specific companies and products) about disclosures, with ProPublica’s Dollars for Docs being updated less frequently but providing greater depth. Payments were obtained for 2013 to 2017. The second generation of _PP_DD reported on USD 9.2 billion in payments from two-thousand companies to nine-hundred thousand physicians. Among _GG_PBT authors with a US affiliation (N = 109), 42.3% had professional degrees that were covered by the Sunshine Act (i.e., PhDs and PharmDs were not covered). The year of publication of each reference for 72 chapters including the Appendix A and Appendix B (N = 3576) was obtained. The publication year of all citations of *Katzung’s Basic and Clinical Pharmacology* (_Kat_BCP, 2018, N = 1777) [23], _Dip_PAPA including eChapters (9^th^ ed., 2017, N = 13,389) [11], and Koda-Kimble and Young’s *Applied Therapeutics: The Clinical Use of Drugs* (_KKY_AT, 2018, N = 15,347) [24], were also obtained for comparison. Remington’s *Science and Practice of Pharmacy* (_REM_SPP) was not included on this measure due to its earlier publication year (2012) [25]. Non-parametric analyses were also completed based on whether citations were ten or more years old.

For the narrative reviews, content experts were consulted. Experts included academic pharmacists, physiologists, molecular biologists, a psychiatry resident, a neuroscientist, a pharmacologist, and a bioinformaticist. Experts were instructed to succinctly provide strengths and limitations and, if applicable, to make comparisons with other textbooks they use. Some provided assessments of a specific chapter, others provided an evaluation of an entire section. Two recent graduates from a Master’s in Biomedical Sciences program provided student perspectives. The goal was not to be comprehensive of all 71 chapters, but instead to provide at least a sampling from most sections.

### 2.2. Data-analysis

Statistical analysis was completed with Systat, version 13.1. Figures were prepared using GraphPad Prism. The sex of contributors of _GG_PBT [22] was compared to other pharmacy and biomedical textbooks obtained previously [8,11]. The age of references was calculated as the difference between publication year and the copyright year. The similarity between CoI databases was evaluated with a Pearson correlation and t-test. Variability was expressed as the SEM with a *p* < 0.05 considered statistically significant.

## 3. Results

### 3.1. Quantitative

In Goodman and Gilman’s *The Pharmacological Basis of Therapeutics* (_GG_PBT) [22], there were 115 authors, of which 32 contributed to more than one chapter. The highest degree was MD for half of authors (47.0%), PhD for two-fifths (45.2%), and the remainder (7.8%) were PharmDs. The preponderance (90.4%) of authors were based in the US.

One-fifth (20.9%) of authors were female—or 19.0% if including multiple chapter contributions. Female authorship in _GG_PBT was equivalent to that of Katzung’s *Basic and Clinical Pharmacology* (_Kat_BCP, 17.0%, *p* = 0.52), but less than Remington’s *Science and Practice of Pharmacy* (_Rem_SPP, 37.0%, χ^2^(1) = 8.84, *p* ≤ 0.003) and Koda-Kimble and Young’s *Applied Therapeutics* (_KKY_AT, 53.0%, χ^2^(1) = 8.84, *p* < 0.001).

**Figure 1:**
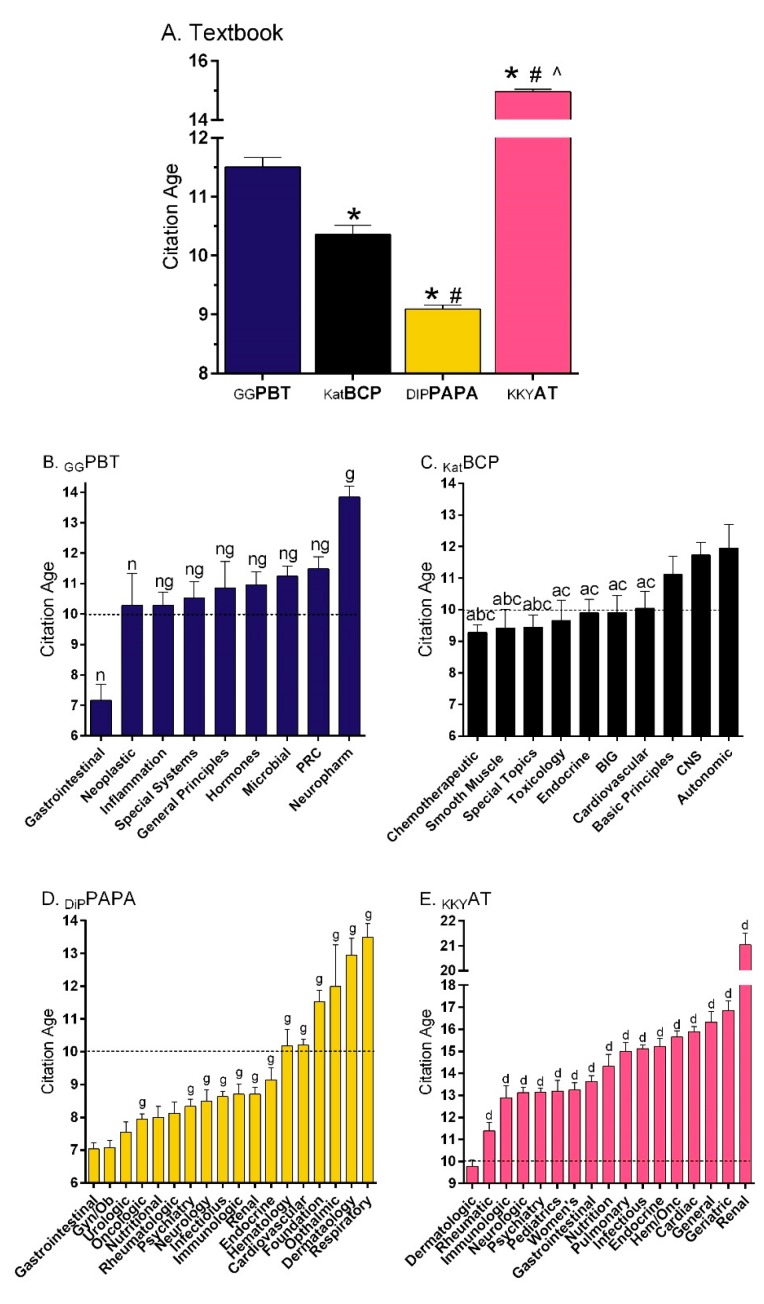
Citation age (± SEM) for Goodman and Gilman’s *Pharmacological Basis of Therapeutics* (_GG_PBT, [22] 13^th^ edition, 2018) relative to Katzung’s *Basic and Clinical Pharmacology* (_Kat_BCP, [23] 14^th^ edition, 2018), DiPiro’s *Pharmacotherapy: A Pathophysiologic Approach* (_DiP_PAPA, [11] 10^th^ edition, 2017), and Koda-Kimble and Young’s *Applied Therapeutics* (_KKY_AT [24], 11^th^ edition, 2018, (**A**). ^*^p < 0.01 versus _GG_PBT, ^#^*p* < 0.0005 versus _Kat_BCP, ^*p* < 0.0005 versus others). Age by section of _GG_PBT (**B**), ^n^*p* < 0.0005 versus Neuropharmacology; ^g^*p* < 0.0005 versus Gastrointestinal), _Kat_BCP (**C**), ^a^*p* < 0.05 versus Autonomic, ^b^*p* < 0.05 versus Basic Principles, ^c^*p* < 0.05 versus Central Nervous System (CNS). PRC: Pulmonary, Renal, Cardiovascular; BIG: Blood, Inflammation, Gout), _DiP_PAPA (**D**), ^g^*p* < 0.01 versus Gastrointestinal Disorders, and Gyncologic and Obstetric Disorders), and _KKY_AT (**E**), Hem/Onc: Hematology and Oncology, ^d^*p* < 0.005 versus Dermatologic Disorders).

Figure 1A shows that the references in _GG_PBT [22] were 12.3% older (11.5 years) than those in _KAT_BCP [23] (10.4, *t*(5321) = 4.42, *p* < 0.0005). The DiPiro’s *Pharmacotherapy: A Pathophysiological Approach* (_DiP_PAPA) [11] citations (9.1 years) were also more recent than _GG_PBT (*t*(16,951) = 15.59, *p* < 0.0001). Similarly, additional analysis determined that more references were at least a decade old for _GG_PBT (49.1%) compared to _Kat_BCP (44.7%, χ^2^(1) = 9.20, *p* ≤ 0.002) or _DiP_PAPA (34.0%, χ^2^(1) = 278.21, *p* < 0.0001). _Kat_BCP also differed from _DiP_PAPA on this measure (χ^2^(1) = 78.44, *p* < 0.0001). However, _GG_PBT had less citations that were ten or more years old relative to _KKY_AT [24] (64.4%, χ^2^(1) = 283.08, *p* < 0.0001). Figure 1B depicts reference age by section of _GG_PBT. The Drugs Affecting Gastrointestinal Function section was significantly newer than all other sections. Conversely, the Neuropharmacology references were 6.7 years older than those for Gastrointestinal, and also significantly less recent than all other sections. There were some broad similarities between _GG_PBT and _Kat_BCP (Figure 1C), with oncology citations being more recent, endocrinology intermediate, and neuroscience the oldest. The gastrointestinal, as well as gynecological, citations were the most recent relative to many other sections in _DiP_PAPA (Figure 1D). The citations in _KKY_AT [24] were significantly older (14.96 ± 0.08) than those of the other three textbooks [10,22,23]. Citations in the dermatologic disorders section (9.77 ± 0.30) were significantly more recent than all other sections, particularly renal disorders (21.04 ± 0.47, *t* (201.03) = 16.36, *p* < 0.0005, Figure 1E) and the eye disorders chapter (27.59 ± 1.05, data in Supplementary Materials).

**Figure 2:**
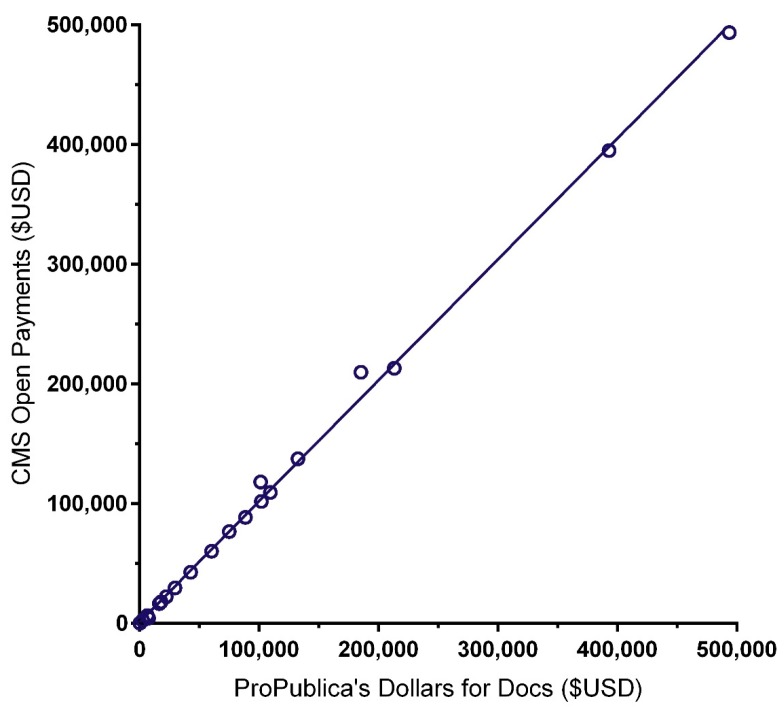
Scatterplot depicting the similarity in undisclosed potential conflicts of interest among Goodman and Gilman’s *The Pharmacological Basis of Therapeutics* (_GG_PBT [22]) authors as reported by ProPublica’s Dollars for Docs (_PP_DD) and the Center for Medicare and Medicaid Service’s (CMS) Open Payments. R^2^ = 0.9978, *p* < 0.0001. Two authors (e.g. oncologist CI = USD 185,223 in DD vs USD 209,943 in OP) had more payments reported by Open Payments.

The similarity of the CoI databases ProPublica’s Dollars for Docs (_PP_DD) and Medicaid Service’s Open Payments (_CMS_OP) was examined with two complementary analyses. The correlation between the total received from 2013 to 2016 (i.e., the most recent available in both databases) was high (*r* (24) = 0.999, *p* < 0.0005; Figure 2). However, the mean amount received was eighteen-hundred dollars higher for _CMS_OP (USD 82,923 ± 24,404) than _PP_DD (USD 81,105 ± 24,122)—a non-significant difference (*t* (25) = 1.59, *p* = 0.13).

The total undisclosed remuneration received by twenty-seven _GG_PBT authors (57.4% of eligible US-based physician-authors, 96.3% male, Minimum = USD 23, Maximum = USD 743,718) from 2013 to 2017 was USD 2.97 million of which almost three-quarters (71.4%) went to the top five authors. The most compensated author received USD 493,536 (66.0% of their total) for royalty or licenses and USD 250,108 (34.0%) for ownership or investment interest. One of the dermatological pharmacology authors received USD 493,536 for ivermectin, a head lice treatment. The psychosis and mania chapter author received USD 238,413 in payments related to two atypical antipsychotics. The oncologist author of “Hormones and Related Agents in the Therapy of Cancer” received USD 228,493 for five breast cancer agents.

Additional analyses were completed on _DiP_PAPA [11] which provides CoI information. Approximately one out of every twelve contributors (20/233 or 8.6%) self-reported a potential CoI. Among the 35 eligible (MD or DO with a US affiliation) authors, 74.3% had a _PP_DD entry (Min = USD 14, Max = USD 729,695, total = USD 1.8 million). However, some discrepancies were identified. A status epilepticus author whose disclosures were self-reported as “none” received USD half-a-million for anti-epilepsy drugs according to _PP_DD. Two authors who did not report disclosures to _DiP_PAPA accepted USD 70–350 thousand in potentially relevant remuneration from 2013 to 2016 (Table 1).

### 3.2. Narrative Reviews

Open-ended evaluations were provided on sixteen chapters, including representation of seven of the nine sections of Goodman and Gilman’s *The Pharmacological Basis of Therapeutics* (_GG_PBT). Table 2 provides strengths and limitations.

#### 3.2.1. General Principles

Chapter 2, “Pharmacokinetics: The Dynamics of Drug Absorption, Distribution, Metabolism, and Elimination”, is a comprehensive and thorough explanation and discussion of the basic concepts of pharmacokinetics. The characteristics and processes of absorption, distribution, metabolism, and elimination are reviewed respectively, including an in-depth discussion of the types of transport across membranes and the influence of pH. The first-pass effect, as well as the benefits and limitations of various routes of drug administration, are also explored. Potentially beneficial additions to the discussion are further examples of specific drugs and their routes of administration, as well as the factors that influence onset of action. There is a brief discussion of rectal administration that could be improved by expanding upon the reasoning behind its incomplete and irregular absorption. Distribution is briefly explored, including a discussion of the differences of distribution among various types of tissues as well as an explanation of protein and tissue binding. While the bone portion of this section focuses mostly on tetracycline antibiotics, an introduction to the pathophysiologic changes in bone that make distribution difficult overall would be helpful, space permitting. The next section on metabolism is a simplistic but comprehensive explanation of phase one and two reactions, as well as first and zero-order kinetics. The excretion section focuses primarily on renal excretion but also includes that by other routes. Learning is aided by highly beneficial figures that depict renal drug handling and a helpful visual of the afferent and efferent arterioles. The final section discusses clinical pharmacokinetics and the “four most important parameters governing drug disposition.” While this section is extensive and includes strong drug examples to explain clinical pharmacokinetics, it could be confusing to the reader that components of “clinical pharmacokinetics” are separated from ADME. It may be beneficial to integrate this section into earlier content. Some examples include moving the discussion of extent and rate of absorption under absorption, clearance under excretion, and volume of distribution under distribution. There is a helpful explanation of maintenance and loading doses, therapeutic window, and dosing intervals along with drug examples with each. However, the explanation of therapeutic drug monitoring is somewhat limited and potentially too generalized. Additional examples of therapeutic drug monitoring (for example, a comparison of therapeutic drug monitoring used for vancomycin, aminoglycosides, and warfarin) would be beneficial to the reader in order to understand the parameters that influence different timing and results of monitoring.

Chapter 3, “Pharmacodynamics: The Molecular Mechanisms of Drug Action” includes a comprehensive and highly detailed discussion of three primary areas of pharmacodynamics including basic concepts, mechanisms of drug action, and signaling pathways. The first section provides a detailed explanation of different receptor types as well as a short discussion of the increased development of biologic agents. The definitions of agonists, antagonists, and their subtypes are provided. A useful addition to this General Principles section would be drug examples of agonists, antagonists, and their subtypes in order to illustrate these concepts further. Specificity of drug receptors and tachyphylaxis are explored, followed by a detailed explanation of affinity, efficacy, and potency. Graphs and mathematical models are used to elaborate upon these concepts. Individual and population pharmacodynamics as well as factors that affect the variability of drug dosing are included. There is also a useful section that expands on the individual patient characteristics that contribute to variability of dosing. There is a section on drug interactions, but it does not mention the effect of CYP enzymes on drug interactions. While this is typically categorized in pharmacokinetics, it may be beneficial to mention their substantial effects on drug levels here. The following section explores the mechanisms of drug action, and expands upon the effects of drugs on ligands, extracellular responses, intracellular pathways, and ions. The mechanisms of anti-infective drugs are also reviewed, which can potentially be eliminated in the overall discussion, or converted to an example instead. There is a helpful review of the structure and function of specific receptor types including second messenger systems and other signaling pathways. Tachyphylaxis and desensitization are discussed again here and could be more beneficial if the discussions on this topic were condensed to one area of the chapter. The chapter ends with an extensively detailed exploration of diseases associated with transcription and translation along with the pharmacotherapies that treat them. Considering that this is an introductory chapter on pharmacodynamics, it may be more useful for this information to be included at a later point in the book. It may be advantageous to consider narrowing this topic to only basic pharmacodynamic concepts. A thorough evaluation of the Pharmacogenomics chapter may be found in Appendix A.

#### 3.2.2. Neuropharmacology

Chapter 15, “Drug Therapy of Depression and Anxiety Disorders”, includes several strengths. Figure 15.1 illustrates detailed information about the variety of antidepressant mechanisms or action. It provides a list of long term cellular regulatory changes in addition to increasing neurotransmitter “dwell time” in the synapse. The development of ketamine and other approaches as novel antidepressants are well described. Information about pharmacokinetics and CYP action is helpful in a practical way to predict, and avoid, drug interaction. This chapter provides a general summary of the basic treatments for anxiety and depression.

As a weakness, chapter 15 does not place very much emphasis on the dopamine or histamine systems/actions in antidepressant effects or depressive etiology. Information about side effects, therapeutic laboratory values, wash-out periods, serotonin syndrome and discontinuation effects are dispersed throughout the text and are not highlighted or emphasized as key pieces of information in designing a treatment strategy. At the beginning of the chapter, depression is immediately categorized into bipolar I and II. It might be more accurate to introduce mood disorders more generally, with an emphasis on depressive symptoms/diagnoses. The authors suggest that there has been limited progress in developing animal models sensitive to antidepressants and anxiolytics. Anxiety is described as “a normal human emotion that serves an adaptive function,” however depression is not.

Another concern is that there are places where information is presented without research citations, such as “Anxious patients appear to be particularly prone to severe discontinuation reactions with certain medications such as venlafaxine and paroxetine; therefore, slow-tapering is required.” The chapter does not include information about options for drug-treatment-resistant symptoms or suggestions for combination approaches [21]. As this is a pharmacology text, that is reasonable. However, it might be helpful to have at least a mention of electroconvulsive therapy or transcranial magnetic stimulation, so readers are better informed of other options if drug treatment has been unsuccessful. More specialized textbooks [21] provide, at least, a nod that the nomenclature for the “Selective” Serotonin Reuptake Uptake Inhibitors is an inaccurate oversimplification [26]. Although recognizing space constraints, greater incorporation of evidence-based psychotherapies like cognitive behavioral therapy, alone and in combination with pharmacotherapies [27,28], is needed.

Chapter 20, “Opioids, analgesia, and pain management”, is well-organized and includes an emphasis on history, receptor signaling, the pathophysiology of pain, tolerance, withdrawal, and medical chemistry for this important drug class. On rare occasions, there is some unusual content and minor areas for reconsideration. Mentioning that opioids do not bind to sigma receptors, twice, may be less useful for new members of the field. The space devoted to specific opioids does not show any simple relationship with their current use patterns [29]. Meperidine and normeperidine, despite concerns with CNS excitation and seizures, are mentioned four times more commonly than the ubiquitous hydrocodone. Tramadol is described, twice, as a “weak opiate agonist”. Katzung’s *Basic and Clinical Pharmacology* (_Kat_BCP) [23] and others have a more nuanced description of the mechanism of action of this agent and the importance of desmethyltramadol [30]. The terms “addict” or “addicts” are used eight times, vs. zero for the less stigmatizing “opioid use disorder” [31].

Chapter 23, “Ethanol”, begins with a brief description about the historical use of alcohol in human civilization as well as epidemiologic overview of problems associated with it; this is quite helpful. There is also a practical overview about alcohol content of different beverages, as well as information about estimating the blood ethanol concentration in end-expiratory alveolar air. Excellent sections with pharmacological properties of methanol and ethanol and its effects are arranged by system. The addition of a Shakespearean quote added emphasis and served as a nice anecdote to illustrate the overview. This is also a good overview of the postulated neurological pathways that are thought to be involved in tolerance and dependence. Descriptions of teratogenicity, genetics, and drug interactions are adequate. Information about treatment of alcohol withdrawal is lacking. No information about choice of drug therapy for withdrawal, or for symptoms triggered therapy protocols (the Clinical Institute Withdrawal Assessment or CIWA protocol for example) for assessment and treatment, is provided. This is a surprising omission as alcohol withdrawal is very prevalent and is associated with considerably increased mortality and morbidity as compared to opioid withdrawal.

#### 3.2.3. Modulation of Pulmonary, Renal, and Cardiovascular Function

Chapter 25 has the challenging task of introducing normal renal structure and function as a basis for the mechanistic understanding of drugs that affect the renal excretory function. The chapter does an incredible job in covering clinically relevant renal concepts. However, it is somewhat arbitrary in what is introduced in the chapter’s introduction as “normal” and what is mentioned later, because it is necessary to mention to understand the mechanics of diuretics and other drugs. Some figures and tables are very helpful (e.g., Table 25-1), while others are overloaded and do not add much value (e.g., Figure 25-2). Overall, it is a good chapter, despite the fact that every pathophysiologist will miss some mechanistic understanding of normal (e.g., Mg^2+^ reabsorption) or abnormal processes (e.g., reduction of urinary Ca^2+^ excretion by thiazide diuretics).

#### 3.2.4. Hormones and Hormone Antagonists

Chapter 48, “Agents Affecting Mineral Ion Homeostasis and Bone Turnover” provides a thorough review of the pharmacological agents used in treating and preventing mineral ion imbalances and bone metabolism. The tables and figures are highly useful and summarize the main points of discussion, particularly the drug summary table at the end of the chapter, which provides a quick reference for understanding the pharmacological agents, their uses, and their clinical effects. This chapter will be useful for both the medical student and seasoned healthcare professional in that, in addition to discussing all of the major pharmacological players involved in treating ion and bone diseases, it makes great attempts to review and contextualize these treatment approaches by providing significant background information. For example, this chapter contains a review of the basic hormonal control mechanisms involved in ion homeostasis, target organs/systems, the relevant bone cell physiology, and a summary of the biology of disorders of mineral homeostasis and bone. The chapter focuses on general concepts that have a high relevance in the clinical setting and are useful for directly understanding the underlying pharmacology. This approach, while on the whole effective, does sometimes come at the expense of missing some detailed but relevant information. For example, the chapter really does not discuss osteoclast function in depth or bone resorption/remodeling effectively, which would aid in the reader’s understanding of drugs like bisphosphonates. This chapter also contains a decent summary discussing the integrated approach to prevention and treatment of osteoporosis. This section does a more than adequate job of generally summarizing the field with respect to the treatment strategies used for managing and preventing osteoporosis.

#### 3.2.5. Gastrointestinal (GI) Pharmacology

Chapters 49 through 51 focus on GI disorders. There are several positive aspects to these chapters. First, the physiology and pathogenesis overview of each chapter and pharmacology of each medication class is brief yet manages to remain quite detailed. The use of figures throughout allows information to be more easily digested. Also, as a new edition, this resource incorporates information on recently approved agents, including medications like eluxadoline (Viberzi^®^) and vedolizumab (Entyvio^®^), which naturally would be omitted by older resources. Finally, information on each drug and drug class appears complete and accurate. Even adverse events recently identified by newer studies, like dementia and chronic kidney disease for proton pump inhibitors (PPIs), are included.

On the other hand, there are several drawbacks as well. This section groups a large number of topics into a small number of chapters, which could make readings challenging to assign to students. For instance, to discuss a topic like *Helicobacter pylori* infection, one must first learn about PPIs early in Chapter 49, then navigate through about two-thirds of the chapter before finally coming across a brief discussion on managing the disorder.

The organization of Chapter 50, which covers motility disorders, emesis, and biliary and pancreatic disease, is particularly hard to follow. The chapter begins by introducing antimotility agents used in small populations and only available through limited access programs, if at all. Meanwhile, very common motility disorders like diarrhea and constipation are buried throughout the lengthy chapter. Also, the complications of cirrhosis, an important GI disorder, are mixed in only in short blurbs and in a confusing manner throughout the chapter. A more complete discussion is vital and its absence is a concerning flaw of this section.

Also, while it may be fitting to limit or omit information on medical interventions, such as fundoplication surgery, relative to a therapeutics textbook, there is limited coverage on nonpharmacologic interventions, which are some of the most common and important recommendations pharmacists can make to patients. Additionally, some aspects of these chapters assume a baseline understanding, which may not be beneficial for students who do not possess this prior knowledge. For instance, terms like distal proctitis are left undefined, yet are used freely when describing the therapeutic role of certain agents. Finally, compared to other resources, this section uses fewer tables, making differences between medications or diseases more difficult to establish.

Overall, chapters 49 to 51 allow individuals to build a strong foundation in the pharmacological treatment of GI disorders by providing a great deal of information in a small number of chapters. However, this section is not perfect. Improvement is needed with regard to organization, coverage of cirrhosis, information on nonpharmacologic interventions, and the use of tables to present data.

#### 3.2.6. Chemotherapy of Infectious Diseases

Chapters 52, 56, 57 and 58 focus on infectious diseases. Chapter 52 is a general chapter focusing on the general principles of antibacterial therapy. There are a few positive aspects to this chapter including the step by step description of which antibiotic type (prophylactic, definitive, suppressive, etc.) should be utilized in the infectious disease (ID) process and detailed explanations of each type. The overview of the pharmacokinetics of antibiotics is very detailed and the use of figures throughout the descriptions provides visual explanations to better understand the information. The explanation on medication resistance is very thorough and ensures that detailed information for the resistance of each medication class does not have to be discussed in each respective chapter.

Chapter 52, on the general principles of antimicrobial therapy, was very informative but does have some disadvantages which make it somewhat difficult to follow. The pharmacokinetics section, though detailed and useful, covers information beyond the need of healthcare providers. The descriptions of E_max_ and K_a_, including equations, are explained well but are not necessary to fully describe the antibiotic classes. This information is not useful when determining what antibiotic to utilize, nor does it explain how dosing is affected based on these values. The explanation of time-dependent and concentration-dependent antibiotics, and antibiotics with a post-antibiotic effect are explained very well; however, a comprehensive table detailing what medications fall into each category is not provided. Most other textbooks include a description of what an infection entails and appropriate selection of antibiotics; however, this chapter jumps directly into the pharmacokinetic information.

Chapters 56–58 discuss various antibacterial agents with descriptions of structure activity relationships, mechanism of actions, antibacterial spectrum, pharmacokinetics and indications for each medication within each respective class. The organization of the chapters has an excellent flow and is consistent from class to class. In chapter 56, the discussion on medications used for urinary tract infections is not consistent with the other medication classes; however, the information provided is very thorough and does incorporate the same information provided by the other class of medications, just in a different manner. Even though there are copious amounts of information provided on each respective class and each medication within the class, the format and explanations are done in a very lay method allowing non-healthcare providers to understand the material as well. The inclusion of historical facts about certain medication classes improves the reading quality and provides background information on either the class or the antibacterial spectrum of the class. In chapter 57, the inclusion of the carbenicillin class is necessary to explain the class as a whole. Other texts will exclude these medications and only discuss piperacillin/tazobactam; however, including the medications not available in the United States, such as ticarcillin and mezlocillin, increases the credibility of piperacillin. Most texts have difficulty explaining the cephalosporin generations; however, chapter 57 does an impeccable job detailing out medications in each generation and relating it back to the antibacterial spectrum. The summary tables at the end of chapters 57 and 58 are all-inclusive and do a superb job at summarizing the chapter.

These chapters are very thorough and useful to a variety of healthcare providers; however, there are a few limitations within these three chapters, which provide information that is not needed or lacks information in certain areas. In chapter 56, when discussing sulfonamides, several medications are referenced which are not commonly used, including one which is not approved for use in the United States. This excess information makes it more difficult to find the relevant information for medications more commonly utilized. One major limitation within these three chapters is the lack of tables throughout the chapter to simplify indications and dosing. All indications are separated into respective paragraphs which makes it more difficult to follow, providing tables after each class highlighting the indication and doses would allow readers to connect all medications within the class. It is understandable that not all new medications could be included due to the publication process being lengthy; however, four medications released within the last year are not included, two of which make a huge impact in practice. Delafloxacin (Baxdela^®^) and the combination of meropenem and vabobactam (Vabomere^®^) were introduced within the last year, but they have not warranted major changes; however, the combination of ceftazidime and avibactam (Avycaz^®^) and the combination of ceftolozane and tazobactam (Zerbaxa^®^), medications introduced specifically to fight off resistant pathogens such a carbapenemase-resistant enterobacteriaceae, are essential for healthcare providers to know about due to increased resistance to other medications in recent years.

Overall, these chapters allow healthcare providers to obtain the necessary pharmaceutics information needed to make decisions in patient care; however, improvements in formatting and the inclusion of newer medications would benefit the text as a whole.

#### 3.2.7. Pharmacotherapy of Neoplastic Disease

Chapter 65, “General Principles in the Pharmacotherapy of Cancer”, is an excellent and reasonably comprehensive background to cancer pharmacology. Strengths of this chapter include: (a) distinguishing slower-growing cancers that have a smaller proportion of actively cycling tumor cells and are thus less responsive to drugs that target the cell cycle; (b) stressing the importance of combinatorial therapy, particularly combinations of molecularly targeted drugs and immunotherapy with more generalized cytotoxic chemotherapy; (c) a discussion of the various challenges of molecular testing to determine those patients for whom specific targeted therapies would be most efficacious. With respect to the last issue, the chapter not only discusses the issue of tumor heterogeneity but also properly cites inherited genetic variation (and not just tumor-specific variation) as a factor affecting treatment response. One criticism is, in the section on resistance, it is stated that the resistant cells pre-exist the treatment which selects for these cells. While this no doubt occurs in the (vast) majority of cases (and the evidence for this for kinase inhibitors is discussed in Chapter 67), it should be noted that resistant cells can occur during treatment as a result of random mutations, unrelated to treatment, that may occur, particularly in tumors exhibiting a hypermutable condition (e.g., MSI+). Also, the much more controversial issue of adaptive mutation, which is starting to attract serious attention from a subset of researchers, could have at least been mentioned in passing. For example, there has been a report of the possible role played by adaptive mutation in the development of resistance to the androgen receptor antagonist bicalutamide in prostate cancer cells [32].

Chapter 66 goes into great detail about several important classes of cytotoxic drugs, mostly those that block cell division and/or promote apoptosis, but also the differentiating agent all-trans retinoic acid (ATRA). Alkylating agents and platinum analogs come in for a particularly extensive review, and one that does justice not only to mechanisms of action and therapeutic efficacy, but also the (often serious) side effects of these agents. The chapter also makes an important distinction between bifunctional agents and monofunctional methylating agents, which is helpful.

Chapter 67, “Pathway-Targeted Therapies: Monoclonal Antibodies, Protein Kinases Inhibitors, and Various Small Molecules”, delves into a detailed examination of pathway-targeted therapies, centered on small molecule inhibitors and monoclonal antibodies, two approaches that are contrasted with respect to range of action and to their side effects (the small molecules tend to have both a greater range of desirable activities as well as negative side effects). Similar to the examination of cytotoxic therapies in the preceding chapter and to the more general introduction of chapter 65, attention is paid to issues of mechanism of action, matching specific therapies to the appropriate type of cancer, including the genetic variation of the cancer, preexisting inherited genetic variation of the patient that affects response, side effects, and the development of resistance. Also, combinatorial therapy is touched upon, an approach particularly useful with the monoclonal antibodies. The sections on angiogenesis inhibitors and immunotherapy (particularly the immune checkpoint inhibitors) was quite good, and this reviewer positively notes the discussion of combinatorial immune checkpoint therapy targeting both cytotoxic T lymphocyte-associated protein 4 (CTLA-4) and programmed death-1 (PD-1). There was some discussion about how to overcome resistance; for example, resistance to the mitogen-activated protein kinase kinase (MEK) inhibitor trametinib can be overcome by combinatorial therapy with the BRAF inhibitor dabrafenib, and there is also discussion about dealing with imatinib resistance. This is all significant; if anything, more on this topic can be included; the same can be said about the ameliorating side effects of these agents. There was some decent discussion on this latter topic; particularly useful was the note about colony-stimulating factors used to deal with hematopoietic toxicity of many anti-cancer therapies. Certainly, an expanded analysis of such topics is always welcomed. The section on histone deacetylase inhibitors was adequate, but there could have been a discussion about butyrate—a fermentation product of dietary fiber—which is a histone deacetylase inhibitor, and one that may significantly mediate the anti-cancer properties of dietary fiber for the colon. These topics could have been broached, which would have also allowed for a discussion of how naturally occurring agents in food may exert preventive action against cancer. Although the book is focusing on pharmacological therapy, the overlap between pharmacology and “medicinal food,” as well as that between prevention and treatment, could have been productively addressed for the sake of completeness. Chemoprevention is a legitimate topic in the field of cancer, both with respect to natural products as well as, perhaps, other pharmacological agents. One issue that could have been included as additional detailed discussion is the approach of inducing apoptosis through hyper-activation (rather than inhibition) of signaling pathways the cancer cell is “addicted to.” There also could have been some discussion of cutting-edge small molecules in clinical trial that target signaling pathways, such as ICG-001-like compounds that inhibit CBP-mediated Wnt signaling. Further, by connecting issues such as targeting signaling pathways, hyperactivating signaling pathways, and molecularly targeted drugs, one could cite the finding that some histone deacetylase inhibitors can hyperactivate Wnt signaling, inducing apoptosis of colorectal cancer cells in culture, pointing to a possible novel therapeutic approach. That underscores one weakness of these chapters—while they tend to give an excellent overview of existing therapies, there is not much about possible novel future approaches.

Chapter 68 offers sound coverage on the role of hormone modulation for cancer therapeutics. Two main approaches were addressed. First, there was discussion about glucocorticoids, which can not only be used for anti-cancer treatment, but also to ameliorate side effects from other forms of cancer therapy. Second, hormone-based therapy for breast and prostate cancer was addressed. Interestingly, while anti-estrogen therapy is now important in breast cancer treatment, in the past, high doses of estrogen were used for certain breast cancers to induce apoptosis. The chapter notes that it is necessary to address the physiological type of breast cancer before commencing therapy, as some forms of the disease are hormone-therapy resistant. The chapter also goes into detail about anti-androgen therapy for prostate cancer, and this is well done, although an analysis of quality-of-life issues would have strengthened the discussion of pharmacology-based treatment options for this disease.

#### 3.2.8. Special Systems Pharmacology

The dermatological and ocular pharmacology chapters have been greatly expanded compared to the 12^th^ edition of _GG_PBT. Five new agents (bentoquatam, coal tar, anthralin, brimonidine, and propranolol) were added to the “miscellaneous agents” section and one agent was removed (podophyllin) in the dermatological chapter compared to last edition. Both chapters have high quality colorful figures of pathways and tables with details such as structural class and efficacy for each agent. Additionally, there is a drug summary table at the end of the chapter with therapeutic uses, clinical pharmacology, and tips for each agent to compliment the text.

The ocular chapter provides excellent explanation of the medications’ mechanisms of action and describes which agent is preferred or which agent provides fewer side effects compared to an agent within the same class. For example, brimonidine is less likely to cause ocular allergy and for that reason, it is more commonly used. Although this information is presented throughout the chapter, it is not accessible in one place. An algorithm highlighting what is first-line and second-line treatment for glaucoma in the ocular chapter would have been beneficial to the reader.

When compared to Koda-Kimble 10^th^ edition [33], the dermatological and ocular chapters in _GG_PBT lack cases. It would have been helpful to have included case questions within the chapter, so the reader can easily see how the pharmacological information is applicable to patients in practice. In comparison to _Kat_BCP [23], the _GG_PBT dermatology chapter reviews more medications. However, the medication section headlines are not as clearly defined and are presented in a less organized format. Within the sunscreen portion in the dermatological chapter, it might have been desirable to have more details about each agent. The chapters use only the generic name of medications. Since pharmacy students are expected to know both brand and generic names for the NAPLEX and in practice, it would have been appropriate to use both within the tables. In contrast, use of brand names would be less useful for medical students. Additional information from a student perspective may be found in Appendix B.

## 4. Discussion

The quantitative and qualitative evaluation provided here is a substantial extension of prior reports [1,2,3,4,5,6,7,8,12]. Evaluating a large (currently >1440 pages) textbook [1,2,3,4,5,6,7] in just a few paragraphs, as is common, loses some nuance and may do a disservice to the breadth of coverage for an interdisciplinary and rapidly changing field. Overall, the editors and authors of _GG_PBT should be recognized for assuming such a large task as each new edition is a substantial and commendable achievement.

Approximately four out of every five _GG_PBT authors were male. Unfortunately, this finding is concurrent with a larger body of evidence [9,10]. For example, a widely used internal medicine textbook had slightly fewer (18.6%) female authors [12]. The gender gap in _GG_PBT authorship showed some improvements relative to the 12^th^ edition where eight out of nine contributors were male [8]. At this rate, _GG_PBT may achieve gender parity when the sixteenth edition is published in 2039. We are not suggesting that editors should recruit authors to achieve an arbitrary numerical representation. However, if the expertise exists, as is evident in some pharmacotherapy textbooks [8,12], and the pool of authors insufficiently reflects this diversity, then the policy that _GG_PBT selects authors would merit reconsideration.

Citations in _GG_PBT were significantly less recent than those found in both _Kat_BCP [23] and _DiP_PAPA [11]. The difference between sections with the most recent and oldest citations was only 2.7 years in _Kat_BCP, but over twice as large for _DiP_PAPA (6.5) and _GG_BCP (6.7). Agents used for gastrointestinal disorders were most recent in _GG_PBT and _DiP_PAPA while drugs acting on the nervous system were the most dated in _GG_PBT and _Kat_BCP. Possibly, the neuropharmacology sections may change with further development of novel agents for Alzheimer’s [34] and neuroendocrine pharmacotherapies for obesity [35]. Citations were oldest, and sections least homogenous, in _KKY_AT [23]. Almost two-thirds of the references in _KKY_AT were one-decade-old or older, suggesting room for updating. Although there is value to referencing the original clinical trials, subsequent editions should strive to also incorporate reviews and systematic reviews.

There is also substantial room for improvement in conflicts of interest transparency in _GG_PBT. With rare exceptions [21], biomedical textbooks do not currently report on CoIs. Even in _Dip_PAPA [11], which discloses self-reported CoIs, four of the top ten highest compensated authors appeared to under-report their disclosures, which could be due to a narrow reporting time frame, a narrow definition of “relevant” CoIs, a threshold, or failing to provide the information to the editors. Primary sources often employ the International Committee of Medical Journal Editors (ICMJE) [36] form for disclosure of potential CoIs where authors are instructed to be inclusive. More specifically, the instructions specify “You should disclose interactions with ANY entity that could be considered broadly relevant to the work. For example, if an article is about testing an epidermal growth factor receptor (EGFR) antagonist in lung cancer, one should report all associations with entities pursuing diagnostic or therapeutic strategies in cancer in general”. On the other hand, the ICMJE instructions might need to be adjusted to incorporate the extended time-frame of textbooks where the same authors commonly contribute to multiple editions over an extended period. Verification of self-reported disclosures with ProPublica’s Dollars for Docs (_PP_DD) and Medicaid Service’s Open Payments (_CMS_OP) would be a trivial task for publishing staff. The recipients of industry-provided misinformation regarding strong opioids likely contributed to the US opioid epidemic [37]. Complete and accurate CoI disclosures will contribute to maintaining the highest degree of trust that content is consistent with the principles of evidence-based medicine [20] in subsequent editions.

Narrative evaluations by content experts revealed several strengths but also some potential for improvement. The neuropharmacology section missed an opportunity to at least begin to acquaint future health care providers that terms in wide-spread use like “mood stabilizers”, “antipsychotics” and “stimulants” are not informative and could be replaced by neuroscience-based nomenclature [25]. There is also a need for more uniform avoidance of stigmatizing language [31].

In general, the chapters on neoplasia are sound, and give a reasonably comprehensive overview on both traditional pharmacological therapies as well as some of the newer, more cutting-edge approaches. There are some weaknesses, however. A better understanding of the basic molecular biology would be helpful; in particular, this would strengthen discussions about the development of resistance to theory, as well as how signaling pathways can be modulated for therapeutic effect. Other areas of improvement would be an increased attention to chemoprevention, novel future approaches, and quality of life issues concerning side effects of treatment. More broadly, identification of which agents are not available in the United States at the time of publication would benefit multiple sections.

Some caveats and limitations are noteworthy. First, the sex differences identified are consistent with a broader pattern in academic medicine [8,9,10,12]. Future research should determine whether ethnic minorities are similarly under-represented as biomedical textbook authors. Second, the CoI databases do not report on non-financial CoIs or the financial relationships of non-physicians (i.e. half of _GG_PBT authors). Therefore, the three million dollars received by authors from pharmaceutical companies is likely an underestimate. The presence of potential CoI, although appreciable, does not necessarily mean that content was impacted in any way. Third, the textbook citation differences in Figure 1A would be less pronounced if _GG_PBT was published slightly (one year) earlier. Therefore, the findings within each textbook (Figure 1B–E) may be more meaningful. On the other hand, while the findings in Figure 1A were hypothesized a priori, the results in Figure 1B–E were not, and may be susceptible to Type I errors. Fourth, although the narrative reviewers came from diverse fields and had substantial experience as educators, health care providers, and biomedical scientists, collectively, we are a far less distinguished group than the esteemed authors of _GG_PBT. There is some subjectivity underlying expert opinion and it is likely that others may have identified somewhat different strengths and limitations in _GG_PBT content.

## 5. Conclusions

This report identifies many strengths of the 13^th^ edition of _GG_PBT [22], but also some areas for improvement including greater diversity of authors, heightened transparency of conflict of interest disclosures, and improved recency of citations in select areas.

## Figures and Tables

**Table 1:** Comparison of self-reported and mandated reporting of conflicts of interest among the ten highest compensated authors of DiPiro’s *Pharmacotherapy: A Pathophysiologic Approach*, 2017 [11]. ^n^ name not listed in the disclosures provided by Access Pharmacy 12/10/18.

Author: Chapter	Self-Report	ProPublica’s Dollars for Docs 2013 to 2016
KD: Parkinson Disease	not reported ^n^	USD 729,695, USD 349,614 for Parkinson Disease treatments (Apokyn: USD 132,702, Azilect = USD 111,326, Duopa: USD 34,934, Deep Brain Stimulation: USD 1200)
JWW: Status Epilepticus	“none”	USD 644,986, USD 533,633 for anti-epileptic drugs (Fycompa: USD 235,680, Oxtellar: USD 78,942, Sabril: USD 62,700, Onfi: USD 58,630, Banzel: USD 49,082, Qudexy: USD 24,122, Aptiom: USD 11,144, Trokendi: USD 10,082, Vimpat: USD 3521)
ESR: Urinary Incontinence	“Consultant: Allergan, Astellas Pharma, and Ferring Pharmaceuticals”	USD 227,510, USD 131,600 for Urinary Incontinence treatments (Botox/Allergan: USD 78,260, Myrbetriq/Astellas: USD 25,584, Toviaz/Pfizer: USD 10,815, Vesicare/Astellas: USD 8790, Interstim/Medtronic: USD 8151)
AM: Multiple Sclerosis	not reported ^n^	USD 99,634, USD 69,907 for Multiple Sclerosis treatments (H.P. Acthar: USD 16,034, Copaxone: 11,591, Plegrity: USD 10,122, Lemtrada: USD 8698, Tysabari: USD 8630, Aubagio: USD 6086; Alemtuzumab: USD 5601, Bateseron: USD 3145)
SSCR: Gastroesophageal Reflux	not reported ^n^	USD 53,588, USD 0 for Gastroesophageal Reflux drugs
DCH: Stroke	not reported ^n^	USD 14,762, USD 125 for Thrombectomy device
DJL: Pulmonary Arterial Hypertension	“none”	USD 13,573, USD 10,817 for Pulmonary Arterial Hypertension treatments (Letaris: USD 4142 Orenitram, USD 2251, Tyvaso: USD 2219, Remodulin: USD 2205)
JP: Pulmonary Function Testing	“none”	USD 2716, USD 0 for Pulmonary Function Testing
MSH: Drug Induced Pulmonary Disease	“none”	USD 2045, USD 0 for Drug Induced Pulmonary Disease
JMC: Colorectal Cancer	“none”	USD 1824, USD 0 for Colorectal Cancer treatments

**Table 2:** Strengths (S) and limitations (L) identified by content experts of Goodman and Gilman’s *Pharmacological Basis of Therapeutics* ([22] 13^th^ edition, 2018). Chapter titles are in italics.

Section I General Principles *Pharmacokinetics*: S: This chapter includes a comprehensive and thorough explanation of the basic concepts of pharmacokinetics. Learning is aided by highly beneficial figures. *Pharmacodynamics*: S: There is a very detailed discussion of three primary areas of pharmacodynamics including basic concepts, mechanisms of drug action, and signaling pathways. L: The drug interactions section does not mention the effect of CYP enzymes. *Pharmacogenetics*: L: A further description of the categories of linkages between genetic variations and drug response phenotypes may be constructive for didactive purposes. Also, navigation links between the drugs discussed and their entries within subsequent chapters may be helpful.
Section II Neuropharmacology: L: An opportunity to introduce the reader to Neuroscience-based Nomenclature [26] for drug classes was missed. *Opioids*: S: This chapter is well organized with an appropriate emphasis on history, receptor signaling, the pathophysiology of pain, tolerance, withdrawal, and medical chemistry. L: Future editions should adopt person-centered language. The repeated reference to only physicians is unfortunate for a book with a broader allied-health audience.
Section III Pulmonary, Renal, and Cardiovascular Function: *Renal*: S: This chapter does an incredible job in covering clinically relevant renal concepts.
Section V Hormones and Hormone Antagonists: *Mineral Ion Homeostasis and Bone Turnover*: S This chapter is useful for medical students and seasoned healthcare professionals.
Section VI Gastrointestinal: S: The physiology and pathogenesis overview of each chapter and pharmacology of each medication class maintains brevity without sacrificing key details. L: The organization of this section could be improved including the addition of more drug comparison tables.
Section VII Infectious Disease *General Principles of Antimicrobial Therapy*: L: The pharmacokinetics section could be better limited to clinically actionable information. *Sulfonamides, Trimethoprim-Sulfamethoxazole, Quinolones*: S: Content is very thorough and accessible for a variety of healthcare providers. L: Medications are discussed which are not commonly used and select newer pharmacotherapies were not discussed. There is an underutilization of tables for indications and dosing.
Section VIII Neoplastic Disease *General Principles in the Pharmacotherapy of Cancer*: S: This is an excellent and reasonably comprehensive background to cancer pharmacology. *Hormones and Related Agents in the Therapy of Cancer*: S: Content about androgen therapy for prostate cancer is well done. L: Greater inclusion of quality of life issues.
Section IX Special Systems: S: The figures and tables are high-quality and nicely compliment the text.

## Supplementary Materials

The following are available online at https://www.mdpi.com/2226-4787/8/1/1/s1.

## Appendix A. Evaluation of the Pharmacogenetic Chapter

*General Principles* further includes Chapter 7, “Pharmacogenetics”, which provides an overview of the terminology and principles that describe the genetic variations that contribute to drug response phenotypes along with a description of the application of individual genotyping data to inform clinical decisions from the knowledge that has been gained through pharmacogenetic studies. The chapter begins by providing concise definitions of pharmacogenetics and pharmacogenomics, which enables readers to readily contrast the differences and similarities of these areas of study. The required task of seeing how to apply the results of pharmacogenomic studies and pharmacogenetic studies in the clinical context is emphasized from here and onward. To appreciate the importance of pharmacogenomics when understanding drug response phenotypes, a description of how we know the overall extent to which genetic variations contribute to changes in drug responses is provided. A highlighted result was the finding, through twin studies, that up to 75% of the variability of drug elimination half-lives was attributed to genetic variations. Such findings are paired with a recognition that there are additional factors that need to be considered in the study of how genetic variation affects drug response. These factors include yet-to-be-characterized interactions between genetic variations with each other and between genetic variations with a variety of environmental factors.

Definitions of drug response phenotypes are subsequently described and how they relate to the terms that are used in genetic studies. For example, changes in pharmacokinetic responses due genetic variation are linked to the terms of the dominant, codominant, and recessive, which characterize phenotypic manifestations of the associated gene variants. An in-depth discussion of the definitions of the different types of genetic variations that can affect drug response is provided. Each type of genetic variation is linked to a description of a change in biological function, which in turn is linked to a change in the drug response. Emphasis is thereby placed on interpreting the genetic variations in terms of the anticipated functional consequences.

With the specific examples of genetic variations that affect drug response via changes in biological activity, an understanding of the wide breadth of the possible mechanisms is gained. As a possible improvement, a further systematic description of the categories of known possible linkages between genetic variations and drug response phenotypes may be provided. For example, the chapter currently describes a delineation of how poor metabolizer and extensive metabolizers are linked to changes in the functional activities of the metabolic enzymes, which constitute part of the pharmacokinetic response upon omeprazole administration. Also, changes in the dosage requirements of warfarin linked to variations in the VKORC1 gene as part of the pharmacodynamic response are described’ and clinical responses are linked to the activation of clopidogrel by CYP2C19. It may be helpful to consider in total such descriptions and provide overall categories of such linkages.

Consider the summary of the linkages provided by Table 1 of [38]. The linkages are described with regard to the overall genetic category (poor metabolizer, extensive metabolizer, therapeutic target etc.), the functional effect (metabolic rate slower than normal, changes in drug efficacy, etc.), the possible drug response phenotype associated with an active drug, and the possible drug response phenotype associated with a pro-drug. With such categorizations, along with examples, the student reader may gain a better understanding of the different types of linkages. For example, in that scheme, one would realize the both clopidogrel and codeine behave in a similar manner in that both are prodrugs that, in the presence of the corresponding enzyme which has relatively high activity, can have high levels that are associated with adverse reactions. In Table 7-2 of the Pharmacogenetics chapter, the gene, drug, and specific drug responses are linked to the categories of drug metabolism and transport, targets and receptors, and modifiers. It is recommended that additional descriptions regarding the different types of drug response phenotypes (such as high toxicity, lack of efficacy, etc.), the type of drug (such as active or prodrug), and the overall functional effect of the genetic variation (such as slower metabolic rate or changes in drug disposition) be added for didactive purposes and to aid with interpretation.

The Pharmacogenetics chapter also advances an understanding of the functional consequences in terms of complete or partial loss of function of the associated gene product. A discussion of the increased effect of variations in genes at positions which are highly evolutionary conserved is provided. Such variations are described as being highly deleterious to biological function and ultimately most likely to affect drug response phenotypes. But an amino acid that is changed, due to a genetic variation, to another residue that is chemically dissimilar can also lead to large changes drug response phenotypes. Such descriptions have been provided explicitly in [38] and in the Pharmacogenetics chapter of the previous (12^th^) edition of Goodman and Gilman’s *Pharmacological Basis of Therapeutics* (_GG_PBT). It is recommended that these delineations be placed back into further editions to clarify that a position which is highly evolutionary conserved may not allow a different amino acid that is chemically similar at the gene product position if that substituted residue does not enable the specific function of the gene product [39]. In the 13^th^ edition of _GG_PBT, the Pharmacogenetics chapter aptly indicates that there are computational tools which predict the functional consequences of variations. These programs are described as using evolutionary conservation and structural information. But, a return to providing a delineation of what these effects are may be helpful.

As a new part of the Pharmacogenetics chapter for the 13^th^ edition, there was an informative discussion regarding the need to consider common versus rare variations when assessing their effects on drug response phenotypes. Discussions are provided on how studies are designed to detect common variations and rare variations using candidate gene association studies and genome-wide association studies. It is made apparent that both rare and common variations would need to be considered to predict the correct dosage requirements of warfarin, for example. The inclusion of the effects of rare and common variants appear to be due in part to research focuses of the contributing authors. For the 12^th^ edition, the authors were Mary V. Relling and Kathleen Giacomini, while the author for the 13^th^ edition was Dan Roden. One research area of these authors is the effects of rare and common variants on drug response phenotypes and clinical care, as illustrated in [40].

Overall, the number and breadth of variations identified in the current Pharmacogenetics chapter provide the reader with an appreciation of the various applications of pharmacogenomic information for predicting drug response phenotypes and informing treatment strategies. But the example drugs are not linked to the full descriptions of their associated physiological and pathophysiological effects that are described in the subsequent chapters of the textbook. A recommended improvement may be to provide navigation hyperlinks to the corresponding descriptions available in the other chapters.

## Appendix B. Evaluation of Two Master’s Students

#1: The new edition of Goodman and Gilman’s *Pharmacological Basis of Therapeutics* (_GG_PBT) [22] seeks to explain the concepts of pharmacology beginning with the basics and continuing through some of the practical applications used in many hospitals today. The organization of the text is superb and allows for easy comprehension. After the basics are covered, the physiological systems and their drug targets are neatly categorized so as to help the reader understand the interactions between the biological system, the underlying disease, and appropriate medication. After each of the tissue or system specific maladies are covered, the text delves into more broad illnesses and their treatments in the infectious disease and chemotherapy sections.

The writing in the text is clear and easy to understand. Each disease is well defined, and the text makes good use of diagrams and figures to explain the mechanisms behind the illness. After elucidating the cause of the problem, the text explains the mechanisms behind each potential treatment, and clearly summarizes their advantages and risks in a table. Any unusual or dangerous interactions of a drug are sufficiently explained, and the precautions to using such a medication are always described.

#2: The 13^th^ edition of Goodman and Gilman’s *Pharmacological Basis of Therapeutics* (_GG_PBT) [22] made the learning and interpretation of pharmacology easier for the reader. The way the content is organized, the reader is better able to pick out important ideas from the support given on each topic. This provides a good outline for studying the material required by any specific course using this textbook.

The order of the text overall offers a lot of information at the beginning, and towards the end there are applications of the reader’s knowledge to the treatment of specific diseases and uses for some of the drugs. The text also provides an explanation for physiological processes before explaining the drugs that affect them, which allows the reader to have a better understanding of the significance and action of the drug. Each chapter is formulated so that the material is interesting, and difficult subjects are explained well so that the reader is not lost in the details. There is sufficient support given for the content, so the reader can apply what they are learning to other outside sources and ideas. Clear and legible figures and tables are placed throughout the text in order for the reader to visualize the content in a different form. Explaining the image well within the text provides a good flow that allows the student to understand the topic better. The drug summary tables that are provided are informational and concise which offers the reader a good summary resource. The antipsychotic molindone is listed as “not available in the US” in Table 16-2 but was listed in October 2018 as available [41]. An online textbook can be regularly updated.

Although the text does engage the reader, _GG_PBT does not provide sufficient critical thinking points throughout the text. There are no problem-solving opportunities for each topic, which limits the application and testing of material. The student would need to purchase yet another book for this [42].
